# Retraction Note: Evaluation of PAX8 expression promotes the proliferation of stomach Cancer cells

**DOI:** 10.1186/s12860-021-00372-8

**Published:** 2021-06-01

**Authors:** Liang-Yu Bie, Ning Li, Wen-Ying Deng, Xiao-Yu Lu, Ping Guo, Su-Xia Luo

**Affiliations:** 1 Department of Oncology, Affiliated Cancer Hospital of Zhengzhou University Henan Cancer Hospital, Zhengzhou, NO. 127, Dongming Road, Jinshui District, Zhengzhou, 450008 Henan China; 2 Department of Pathology, Affiliated Cancer Hospital of Zhengzhou University Henan Cancer Hospital, Zhengzhou, 450008 Henan China; 3 Department of Oncology, The First Affiliated Hospital of Nanyang Medical College, Nanyang, 473061 Henan China

**Retraction Note: BMC Mol Cell Biol 20, 61 (2019)**

**https://doi.org/10.1186/s12860-019-0245-9**

The authors have retracted this article because it has been published previously [1]. Su-Xia stated on behalf of all co-authors that they agree with this retraction.
